# Supplementary material for: Salmonella Typhimurium biofilm disruption by a human antibody that binds a pan-amyloid epitope on curli
Source: Nat Commun. 2020 Feb 21;11:1007. doi: 10.1038/s41467-020-14685-3 (PMC7035420; doi:10.1038/s41467-020-14685-3)
Supplement: Supplementary file 1 — Supplementary Information [file 41467_2020_14685_MOESM1_ESM.pdf]

SUPPLEMENTARY INFORMATION

Tukel et al.

Antibody	Oligomers nM	Fibrils nM	CTB oligo nM	CTB mono nM	NTB mono nM
3H3	40	2.1	35	3.4	240
4G1	8.8	14	11.4	16	1700
4A6	1300	11	>2000	16	>2000
2C10	>2000	16	>2000	>2000	>2000
6E10	69	56	50	47	158

Supplementary Table 1. MAb binding to Aβ42 conformers measured by Surface Plasma Resonance (SPR). Calculated affinity constants (K_D) were obtained using Qdat software. Oligomers, ADDLs/globulomers; CTB oligo, C-terminal biotinylated ADDLs/globulomers; CTB mono, C-terminal biotinylated monomers; NTB mono, N-terminal biotinylated monomers. 6E10, mouse mAb to Aβ amino acids 1-16 (BioLegend, San Diego, CA). Compared to the the N-terminal biotinylated monomer, all of the mAbs exhibit lower affinity binding to some or all of the Aβ42 oligomers or fibrils. The 3H3 binding data is qualitatively most similar to the 4G1 and 6E10 mAbs. 4A6 and 2C10 preferentially bind Aβ fibrils.

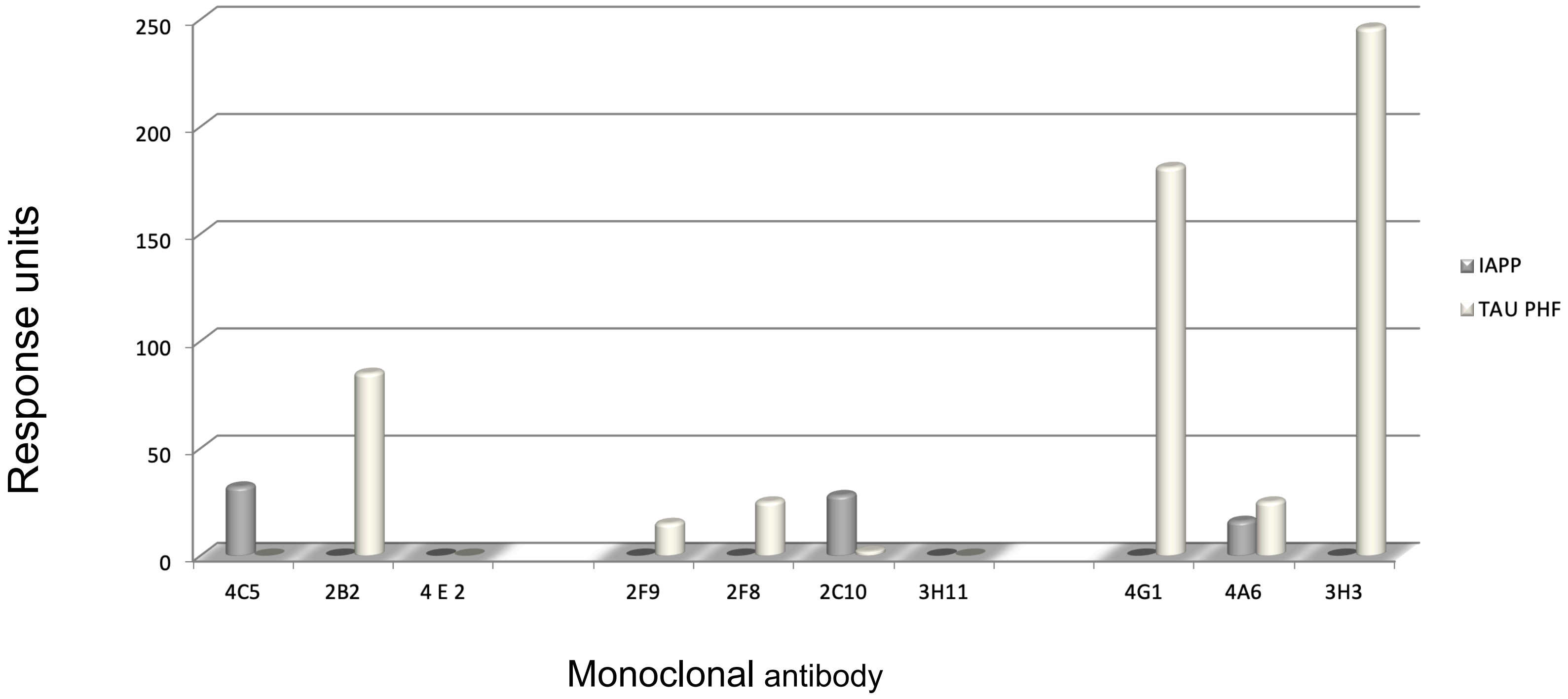

Supplementary Figure 1. Human mAbs were assayed by SPR for binding to aggregated islet amyloid peptide IAPP and tau paired helical filaments (PHF) isolated from AD brain homogenates. The antibody concentrations used in this study were 110 nM for the IAPP and 66.7 nM for the tau-PHF. The 3H3 and 4G1 mAbs are notable for binding to Tau PHF, consistent with recognition of a pan-amyloid epitope. The additional mAbs tested are human IgGs isolated from the same patient as 4G1.

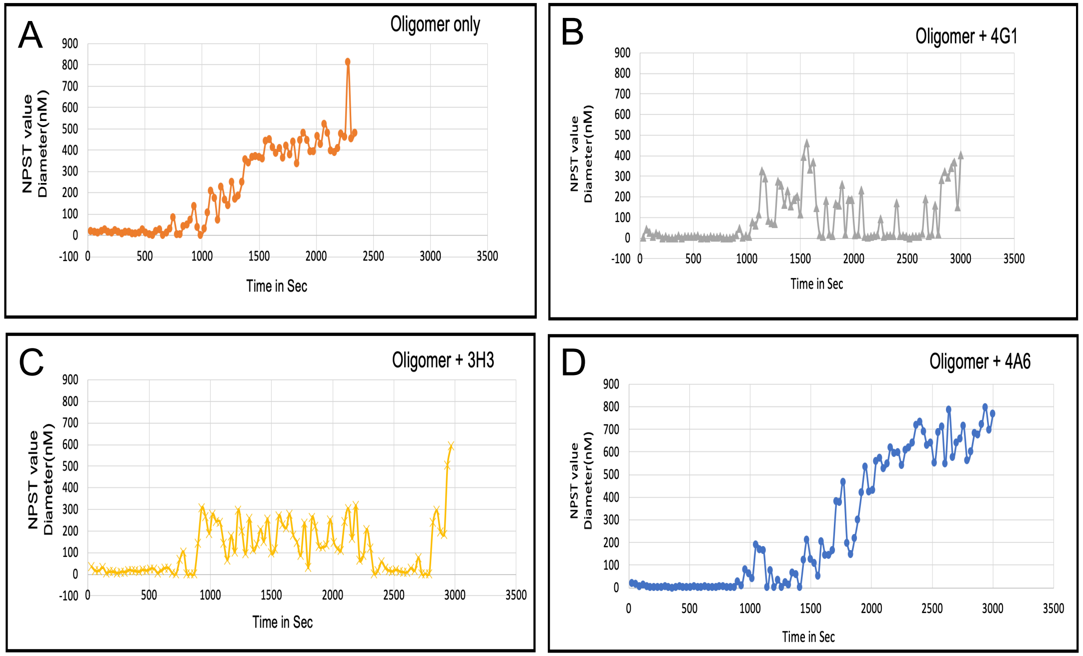

Supplementary Figure 2. Fibrillization of **Aβ42** of amyloid beta oligomers alone (**A**) or in presence of anti-amyloid mAbs, 3H3 (B), 4G1 (**C**) and 4A6 (**D**).

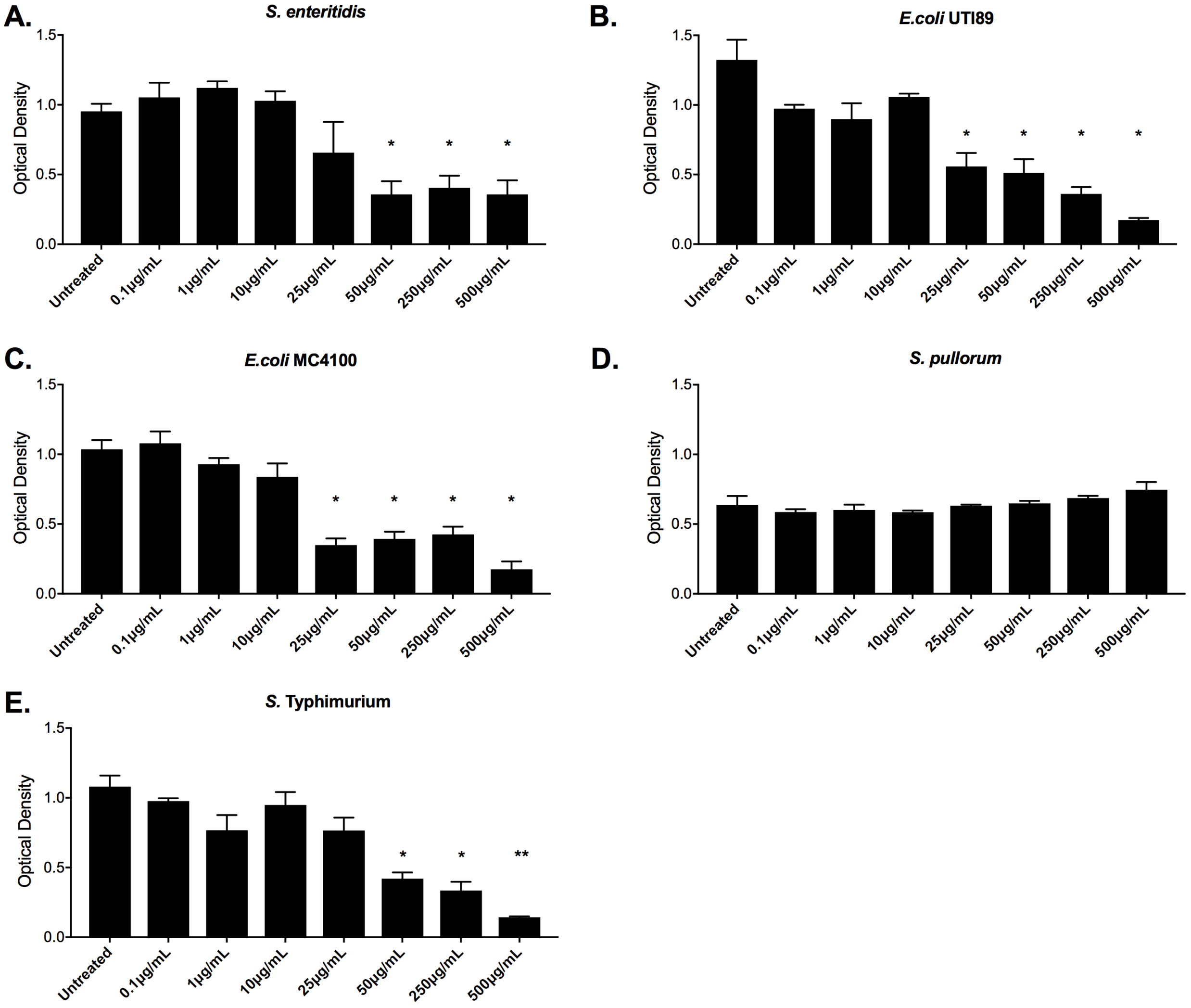

Supplementary Figure 3. 3H3 disrupts biofilm formation in clinical isolates of *Salmonella* and *E. coli*. Biofilms of **A.** *S. enteritidis*, **B.** *E. coli* UTI89 **C.** *E.coli* MC4100, **D**. *S. pullorum*, and **E.** *S.* Typhimurium were grown in the absence (untreated) of presence of 3H3 antibody (0.1μg/mL, 1μg/mL, 10μg/mL, 25μg/mL, 50μg/mL, 250μg/mL and 500μg/mL). After 72 hours, biofilms were stained with crystal violet, and the optical density at 570 nm was determined. Mean and SE were calculated from results from at least two independent experiments. * $p < 0.05$, ** $p < 0.01$ as determined by ANOVA with Tukey post hoc analysis.

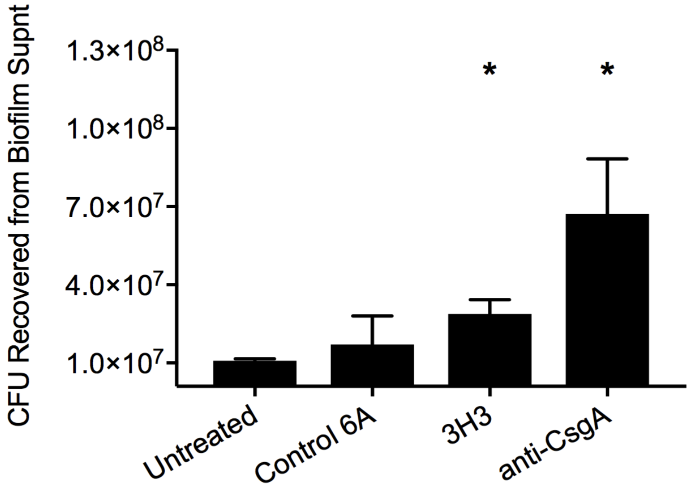

Supplementary Figure 4. 3H3 enhances the number of bacterial cells recovered from biofilm supernatants. *S.* Typhimurium biofilms were incubated in the absence of mAb (untreated) or in the presence of 0.5 mg/ml control A6, 0.5 mg/ml 3H3, or anti-CsgA serum. After 72 hours, 100 µl biofilm supernatant was collected, and the bacteria were enumerated as colony forming units. Mean and SE were calculated from results from at least three independent experiments. * p <0.05, ** p <0.01 as determined by Student's t-test.

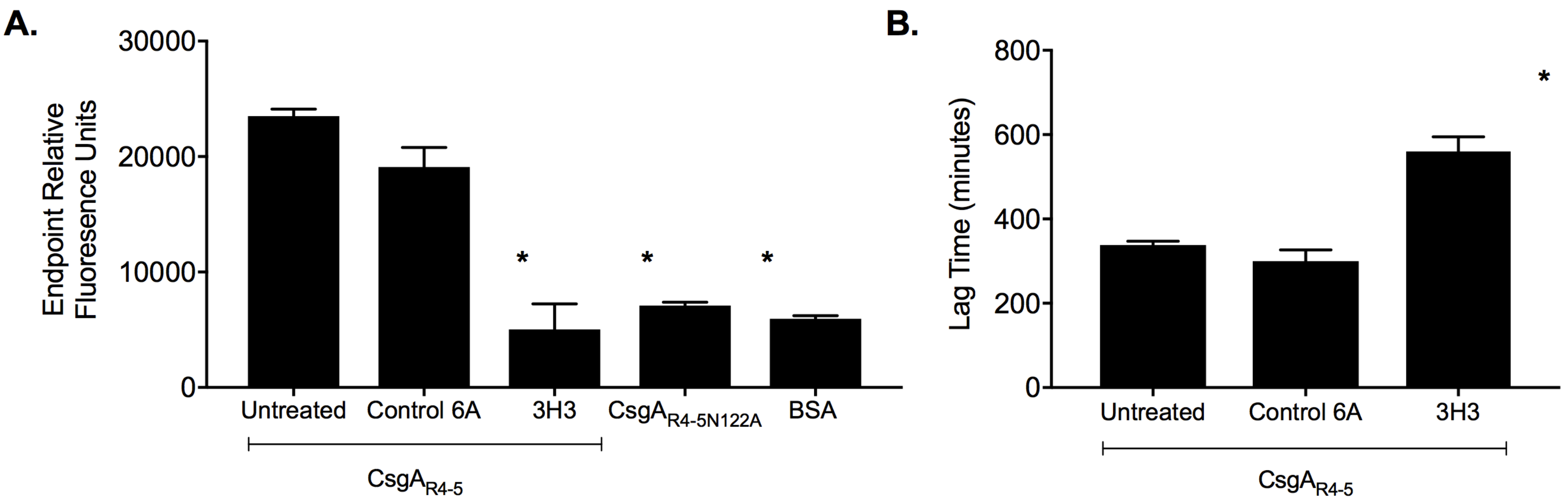

Supplementary Figure 5. 3H3 inhibits CsgA fibrillization A. Samples of 100 μM $CsgA_{R4\text{-}5}$, 100 μM $CsgA_{R5\text{-}4N122A}$, or 500 μg/ml BSA were mixed with 10 μM ThioflavinT (ThT) in the presence of either 0.5 mg/ml 3H3 or 0.5 mg/ml control A6, and fluorescence of ThT (excitation 440 nm/emission 490 nm) was monitored. Endpoint RFU is plotted. **B**. Lag times of fibrillization of $CsgA_{R4\text{-}5}$ in the presence of control 6A or 3H3. Mean and SE were calculated from results from at least three independent experiments. * p <0.05, ** p <0.01 as determined by Student's t-test.

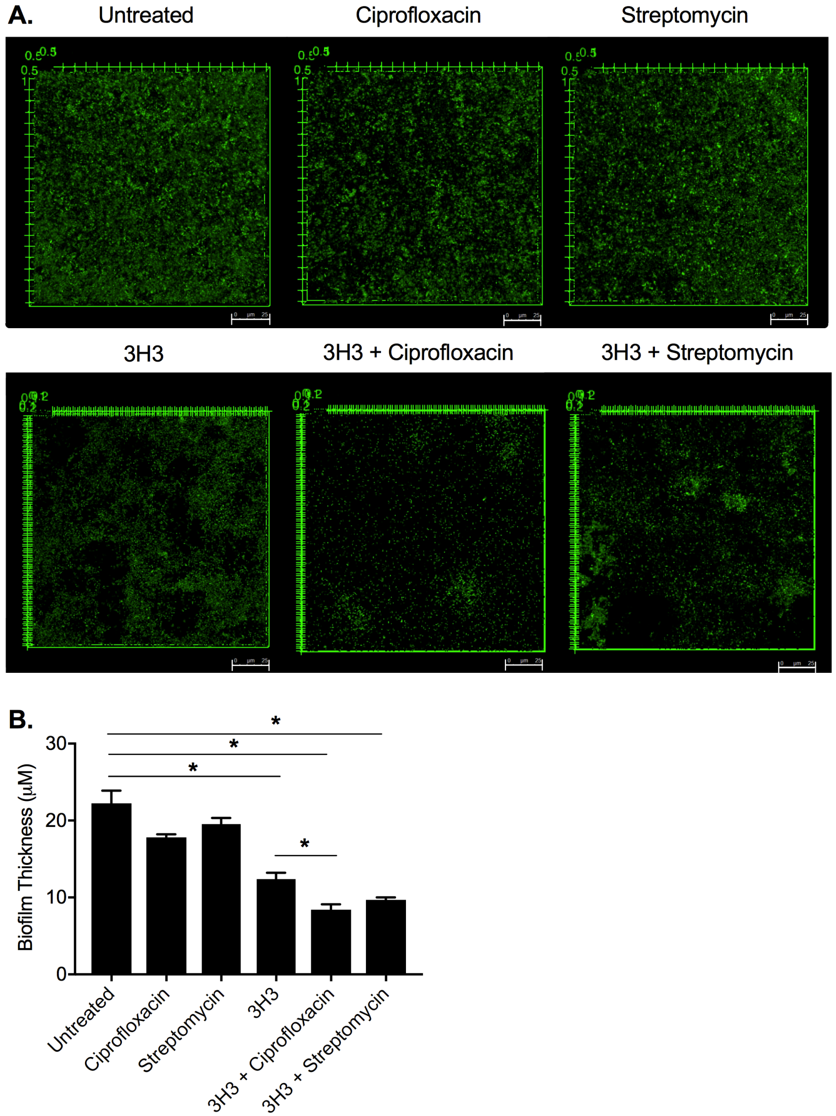

Supplementary Figure 6. Synergistic effect of 3H3 and antibiotic treatment. A. *S.* Typhimurium biofilms were untreated or were incubated in the presence of 0.5 mg/ml 3H3 for 72 hours. After 72 hours, biofilms were subjected to treatment with 0.125µg/ml ciprofloxacin or 12.5µg/ml streptomycin for an additional 24 hours. Biofilms were stained with Syto9 (green), washed extensively, and visualized using Leica TCS confocal microscopy at 63x. Scale bars represent 25 µm. ImageJ was used to create 3D reconstructions of z-stacks**. B.** Biofilm thickness (µm) was determined from z-stacks using Leica TCS software. Mean and SE were calculated from results from at least two independent experiments. * $p < 0.05$
