# Supplementary material for: Salmonella Typhimurium biofilm disruption by a human antibody that binds a pan-amyloid epitope on curli
Source: Nat Commun. 2020 Feb 21;11:1007. doi: 10.1038/s41467-020-14685-3 (PMC7035420; doi:10.1038/s41467-020-14685-3)
Supplement: Supplementary file 3 — Description of Additional Supplementary Files [file 41467_2020_14685_MOESM3_ESM.pdf]

Description of Additional Supplementary Files

File Name: Supplementary Movie 1

Description: *S.* Typhimurium biofilm was formed in the absence of mAb (untreated) or in the presence of 0.5 mg/ml control antibody A6 and treated with 10 µL Crimson FluoSpheres (red). After incubation for 72 hours biofilms were stained with Syto9 (green) and bead movement was captured using Leica TCS confocal microscopy at 63x.

File Name: Supplementary Movie 2

Description: *S.* Typhimurium biofilm was formed in the absence of mAb (untreated) or in the presence of 0.5 mg/ml 3H3 and treated with 10 µL Crimson FluoSpheres (red). After incubation for 72 hours biofilms were stained with Syto9 (green) and bead movement was captured using Leica TCS confocal microscopy at 63x.

File Name: Supplementary Movie 3

Description: *S.* Typhimurium biofilm was formed in the absence of mAb (untreated) or in the presence of 0.5 mg/ml anti-CsgA serum and treated with 10 µL Crimson FluoSpheres (red). After incubation for 72 hours biofilms were stained with Syto9 (green) and bead movement was captured using Leica TCS confocal microscopy at 63x.
